# Lengthening of Bones

**Published:** 1889-04

**Authors:** Marcell Hartwig


### *LENGTHENING OF BONES.*

Progress in science bases upon proper induction from previously established facts. It is hardly one year that the reporter proposed lengthening of the condyle in genu varum respectively valgum by instigating its growth through an inserted ivory nail, (*Medical Record*, March 24, 1888, page 328,) and already Schüller comes forward with a very analogous system and practical achievements of great extent. He united all previously known measures for stimulating the growth of bones in a system. He uses the ivory nail, the elastic constriction (Dumericher and Helferich,) above the bone, (sufficient to compress the veins, but not the arteries,) the downward massage, gymnastic exercise, a diet containing plenty of lime, and reduces the liability of lactic acid being formed in the stomach. Sea-bath he praises warmly. In two cases of essential paralysis of children and retarded growth of one leg, he obtained, with these measures (without nails—nightly sleeping with the elastic constriction) a lengthening of the shortened extremity amounting to one centimeter in three months. To come to a case of double genu valgum, he obtained an almost perfect restitution by stimulating both external condyles (all other measures were included, of course,) by the insertion of nickel-plated steel nails, antiseptically, for five days, two fingers' breadth above the epiphyseal cartilage. The reporter is pleased to see such an early proof of the correctness of his proposition, and wishes to state, further, that Schüller's opinion is that the nearer the nail is inserted above the epiphyseal cartilage, the more intense effect is to be expected, although the cartilage itself is to be avoided, of course. Schüller's argument is correct, as it is well known that destruction of the epiphyseal cartilage interrupts the growth *in locu.* Should the nail accidentally prick the epiphyseal cartilage, the reporter ventures to believe that it would, nevertheless, do no harm, as perforating the cartilage antiseptically is not destructive.—MARCELL HARTWIG.

